# A Case of Third Dentition

**Published:** 1869-04

**Authors:** H. H. Nelles

**Affiliations:** Dentist


A Case of Third Detention.—Dr. H. H. Nelles, dentist, reports in the *Canada Journal of Dental Science*, the case of a lady 45 years of age, from whom he extracted a number of roots preparatory to the insertion of a full set of artificial dentures. Upon a second visit, to his surprise, he found that nature had sent forth a well developed superior *cuspidatus*. In anticipation that nature would complete the work thus auspiciously begun, Dr. Nelles deferred inserting an artificial set of teeth. This case brings to mind a fact related of himself, by a gentleman of our acquaintance. When he was nineteen years of age, his four upper incisors were broken off by a blow with an iron bar. The roots were extracted but no artificial teeth inserted. Some months after, while at the table eating, he was greatly startled to discover the points of teeth projecting through the gum. The incisors soon fully supplied the place of the lost second teeth, and are as perfect in every particular as one could wish.—Jr. *in Pac. Med. and Surg. Journal.*

